# Sensitivity, Specificity and Predictive Value of Heart Rate Variability Indices in Type 1 Diabetes Mellitus

**DOI:** 10.5935/abc.20170024

**Published:** 2017-03

**Authors:** Anne Kastelianne França da Silva, Diego Giuliano Destro Christofaro, Aline Fernanda Barbosa Bernardo, Franciele Marques Vanderlei, Luiz Carlos Marques Vanderlei

**Affiliations:** 1 Universidade Estadual Paulista Júlio de Mesquita Filho (UNESP), Presidente Prudente, SP - Brazil; 2 Faculdade de Educação e Cultura de Vilhena, Vilhena, RO - Brazil

**Keywords:** Heart Rate, Diabetes Mellitus, Type 1, Predictive Value of Tests, Sensitivity and Specificity, Autonomic Nervous System

## Abstract

**Background:**

Heart rate variability (HRV) indices may detect autonomic changes with good diagnostic accuracy. Type diabetes mellitus (DM) individuals may have changes in autonomic modulation; however, studies of this nature in this population are still scarce.

**Objective:**

To compare HRV indices between and assess their prognostic value by measurements of sensitivity, specificity and predictive values in young individuals with type 1 DM and healthy volunteers.

**Methods:**

In this cross-sectional study, physical and clinical assessment was performed in 39 young patients with type 1 DM and 43 young healthy controls. For HRV analysis, beat-to-beat heart rate variability was measured in dorsal decubitus, using a Polar S810i heart rate monitor, for 30 minutes. The following indices were calculated: SDNN, RMSSD, PNN50, TINN, RRTri, LF ms^2^, HF ms^2^, LF un, HF un, LF/HF, SD1, SD2, SD1/SD2, and ApEn.

**Results:**

Type 1 DM subjects showed a decrease in sympathetic and parasympathetic activities, and overall variability of autonomic nervous system. The RMSSD, SDNN, PNN50, LF ms^2^, HF ms^2^, RRTri, SD1 and SD2 indices showed greater diagnostic accuracy in discriminating diabetic from healthy individuals.

**Conclusion:**

Type 1 DM individuals have changes in autonomic modulation. The SDNN, RMSSD, PNN50, RRtri, LF ms^2^, HF ms^2^, SD1 and SD2 indices may be alternative tools to discriminate individuals with type 1 DM.

## Introduction

Type 1 diabetes mellitus (DM), an autoimmune disease that results from the destruction of pancreatic beta cells with consequent insulin deficiency,^1,2^ has affected an increasing number of individuals in the world at younger ages.^3^ Every year, approximately 15 thousand children are diagnosed with type 1 DM and 3,700 children with type 2 DM.^4^

Type 1 DM patients may have autonomous nervous system (ANS) dysfunction, which may be identified by heart rate variability (HRV) analysis.^5,6^ HRV is a simple, accessible, non-invasive method that describes oscillations between consecutive heartbeats (RR intervals, RRIs), which are associated with the effects of ANS on sinus node.^7^

Analysis of HRV has shown that individuals with type 1 DM have reduced overall variability as compared with healthy subjects of different ages.^8-11^ Besides, parasympathetic loss with sympathetic override^12^ and reduced magnitude and complexity of HRV^13,14^ have been reported in these individuals.

HRV has been used to identify autonomic changes and, despite studies showing its efficacy in the clinical practice in different populations, the use of HRV for this purpose is still incipient. In this context, studies have indicated that some HRV indices can detect autonomic changes with relative sensitivity and describe changes in cardiac rhythm with good diagnostic and prognostic value.^15,17^

In middle-aged adults with type 2 DM, Khandoker *et* al.^15^ found that the SD1 (standard deviation of the instantaneous beat-to-beat variability) index, extracted from the Poincaré plot, and the SampEn (sample entropy) can identify cardiac autonomic dysfunction with the best diagnostic accuracy. The authors also showed that the HRV may have a practical diagnostic and prognostic marker in this population.

However, in type 1 DM patients, studies of this nature are still scarce, since most of them provides a comparison of HRV between subjects with and without DM, without analyzing the discriminatory power of these indices. Such studies would not only provide new information on the theme, but also determine HRV indices with the best diagnostic and prognostic value in these individuals. This would allow a better risk stratification, and elaboration of preventive programs and new therapeutic strategies for these patients.

In light of this, the aim of this study was to compare HRV indices and evaluate their sensitivity, specificity and predictive value in young type 1 diabetic patients and healthy volunteers. We hypothesize that changes in autonomic behavior in young subjects with type 1 DM can be identified by HRV analysis and that this is an effective diagnostic and prognostic marker in this population.

## Methods

### Patients

Patients with diagnosis of type 1DM were recruited from the database of community health centers and by contact with endocrinologists in Presidente Prudente, Brazil, and healthy volunteers were recruited from a public university of the same city. Sample size calculation was performed based on the RMSSD (square root of the mean of the squares of successive differences between normal RRI). Considering a magnitude of the difference of 19.85, standard deviation of 25,30,^18^ and alpha and beta risk of 5% and 80% respectively, the sample size calculated was 25 individuals per group.

A total of 88 volunteers of both sexes, aged between 18 and 30 years were recruited and allocated into two groups: type 1 DM group, composed of 43 young type 1 DM patients (20 men and 23 women, mean age of 21.82 ± 5.07 years, time of diagnosis of 11.20 ± 6.01 years), and control group, composed of 45 young healthy volunteers (21 men and 24 women; mean age of 21.35 ± 2.82 years).

Inclusion criteria were age between 18 and 30 years, clinical diagnosis of type 1 DM confirmed by blood test and medical records (for type 1 DM group), and individuals with cardiorespiratory diseases, smoking habit, or alcoholics were excluded. Six volunteers with RRI time series with a sinus beat < 95%^19^ were excluded.

All subjects were informed about the objectives and procedures of the study, and those who agreed to participate signed an informed consent form before being included in the study. All procedures were approved by the Ethics Committee of the School of Science and Technology of UNESP, Presidente Prudente campus (report number 417.031).

### Data collection

Data were collected in a temperature (21ºC-23ºC) and humidity (40%-60%) controlled room, in the afternoon period from 13h and18h to minimize the influence of the circadian rhythm.^20^ For individual assessments, patients were instructed to abstain from alcohol and autonomic nervous system stimulants, such as coffee, tea and cocoa in the 24 hours before the study day.

All volunteers were assessed using a protocol that included 'identification' - age, sex, time of diagnosis (for DM group) and use of drug therapy, 'physical examination', 'clinical evaluation', and 'autonomic assessment, in this order. Physical and clinical evaluation included the assessment of cardiovascular and body composition parameters, physical activity level and postprandial glycemia.

### Physical and clinical assessment

Systolic arterial pressure (SAP) and diastolic arterial pressure (DAP) were measured on the left arm, in patients in sitting position, using a stethoscope (Littman, Saint Paul, USA) and aneroid sphygmomanometer (Welch Allyn - Tycos, New York, USA), according to the VI Brazilian Guidelines for Arterial Hypertension.^21^ Heart rate (HR) was determined using the Polar S810i monitor (Polar Electro, Kampele, Finland).

Anthropometric measurements and body fat percentage were determined in all volunteers. Body weight was measured using a digital scale (Welmy R/I 200, Brazil), height was determined using a stadiometer (Sanny, Brazil), and body mass index (BMI) (weight/height^2^, kg/m^2^) was calculated according to the Brazilian Guidelines for Obesity.^22^ Waist circumference (narrowest abdominal perimeter between the lowest ribs and the iliac crest) and hip circumference (widest part of the gluteal region at the level of the great trochanters) were measured with an inelastic tape (Sanny, Brazil), and the waist/hip ratio (WHR) were calculated.^23^

Percentage of body fat was measured by bioelectrical impedance analysis (Maltron BF 906 Body Fat Analyser).^24^ The level of physical activity was determined by the short version of the International Physical Activity Questionnaire (IPAQ).^25^ For random glucose test, a drop of blood from a finger prick was placed on a One touch ultra test strip (Johnson & Johnson Medical, Brazil) and analyzed by its glucometer. All participants were free to eat, and fasting was not required for the tests.

### Autonomic assessment

After initial instructions, a chest strap was placed on the distal third of the sternum, and the Polar S810i HR monitor was placed on the wrist (Polar Electro, Finland). This instrument has been previously validated for detecting beat-to-beat heart rate variability.^26,27^ Then, the volunteers were placed on a bed in dorsal decubitus position, and instructed to breath spontaneously remain at rest, yet awake, for 30 minutes, and avoid conversation. After data collection for autonomic modulation analysis, the subjects were allowed to leave the room.

For HRV analysis, beat-to-beat heart rate was recorded during all the experiment. One thousand consecutive RRIs were selected from the highest signal stability period by digital filtering^28^ (using the Polar Precision Performance SW software, version 4.01.029 with a moderate filter), complemented by manual filtering to eliminate premature, ectopic beats and artifacts. Only series with more than 95% of sinus beats were included in the study.^19^ By a visual analysis, there were no artifacts or ectopic beats that could affect the HRV analysis. For analysis of HRV, time- and frequency-domain linear indices, geometric indices and nonlinear indices were used.

Time-domain analysis^7^ was performed by SDNN (standard deviation of normal RR intervals), RMSSD and PNN50 (percentage of adjacent RRIs that differ by more than 50 ms). For frequency-domain analysis,^7^ low-frequency (LF: 0.04 - 0.15 Hz) and high-frequency (HF: 0.15 - 0.40 Hz) spectral components (ms^2^ and normalized unit) were used, as well as the LF/HF ratio). Spectral analysis was calculated using the fast Fourier Transform algorithm.

The triangular index and TINN (triangular interpolation of RR intervals) were calculated using the density histogram of normal RRIs, which displayed all possible RRIs values in the horizontal axis and their frequencies in the vertical axis. The connection of the midpoints of each column of the histogram generates a triangular figure, from which these indices were extracted. Both triangular index and TINN values express the global ANS condition.^29^

The nonlinear indices used for the analysis were the Poincaré plot and the approximate entropy (ApEn). The Poincaré plot is a time series graphic representation which plots each RRI against its previous interval.^29^ The plot was analyzed by using the following indices: SD1, SD2 (long term variability of continuous RRIs) and SD1/SD2 ratio.^7^ ApEn describes the RRI complexity; it measures the regularity and the logarithmic probability that the time series patterns remain similar for all comparisons. The greater the ApEn value, the higher the RR series complexity.^30^

All indices were calculated by the HRV analysis software, version 2.0^31^ (Kubios, Biosignal Analysis and Medical Image Group, Department of Physics, University of Kuopio, Finland).

### Data analysis

First, data normality was tested using the Shapiro-Wilk test. Between-group comparisons were performed by the independent t test (for parametric data) or the Mann-Whitney test (for nonparametric test). Data with Gaussian distribution (height, HR, WHR and body fat percentage) were expressed as mean and standard deviation, whereas data whose normality was not confirmed (age, weight, BMI, SAP, DAP, glycemia and weekly physical activity) were presented as median and interquartile range. Between-group comparisons of HRV data were performed by covariance analysis, adjusted by confounding factors (BMI and random glucose levels).

The HRV cutoff points were defined by the Receiver Operating Characteristic (ROC) curve. The sensitivity, specificity, positive predictive value and negative predictive value for the occurrence of events were also determined. An area under the curve ≥ 0.650 was considered significant.^17^

The level of significance was set at 5%. Statistical analysis was performed using the Statistical Package for the Social Sciences, version 15.0 (SPSS Inc., Chicago, IL, EUA) and MedCalc Software bvba, version 14.10.2 (Ostend, Belgium).

## Results

Data of 88 volunteers (43 type 1 DM patients and 45 healthy controls) were assessed. Six volunteers were excluded because of errors in RRIs greater than 5%, and the final sample was composed of 39 young subjects with type 1 DM (19 men and 20 women) and 43 young healthy controls (21men and 22 women).

Table 1 describes general characteristics of both groups. Subjects with type 1 DM had higher body mass, BMI, HR, random glucose levels and % body fat than controls (p < 0.05). All diabetic individuals were insulin-dependent, and 15 (38.46%) used additional medications, other than insulin - five (12.82%) used antihypertensive agents, eight (20.51%) for thyroid diseases, three (7.69%) for cholesterol control, five (12.82%) used contraceptive agents, and eight for other conditions, including rhinitis, diabetic polyneuropathy, peripheral neuropathy and epilepsy.

**Table 1:** Characteristics of diabetes mellitus and control groups

Variables	Control (43)	Type 1 DM (39)	p value
Age^b^ (years)	21.00 (5.00)	21.00 (7.00)	0.534
Body mass^b^ (kg)	60.30 (22.80)	68.15 (22.90)	0.013
Height^a^ (m)	1.69 (0.09)	1.73 (0.17)	0.461
BMI^b^ (Kg/m^2^)	22.19 (4.67)	24.19 (5.84)	0.011
WHR^a^ (cm)	0.77 (0.06)	0.80 (0.10)	0.102
SAP^b^ (mmHg)	110.00 (20.00)	110.00 (10.00)	0.757
DAP^b^ (mmHg)	70.00 (10.00)	60.00 (10.00)	0.620
HR^a^ (bpm)	70.76 (10.04)	80.00 (16.00)	0.000
Random glycemia^b^ (mg/dl)	93.00 (20.00)	162.00 (168.00)	0.000
Body mass^a^ (%)	21.86 (7.58)	26.00 (9.60)	0.044
Weekly physical activity^b^ (minutes)	320.00 (440.00)	280.00 (510.00)	
Time of diagnosis^a^	---	11.71 (5.99)	---

a mean (standard deviation);

b median (interquartile range). Type 1 DM: type 1 diabetes mellitus; BMI: body mass index; WHR: waist-hip ratio; SAP: systolic arterial pressure; DAP: diastolic arterial pressure; HR: heart rate.

Table 2 shows linear and nonlinear values of HRV of both groups. Diabetic subjects had significant lower values of DNN, RMSSD, PNN50, RRTri, LF ms^2^, HF ms^2^, SD1 and SD2.

**Table 2:** Indices of heart rate variability in diabetes mellitus and control groups adjusted by body mass index and random glucose levels

Index	Controls (n = 43)	Type 1 DM (n = 39)	p value
SDNN	66.97 (22.17)	41.99 (19.65)	0.000
RMSSD	55.59 (21.60)	32.73 (17.43)	0.000
PNN50	33.64 (19.97)	14.79 (15.68)	0.000
TINN	220.81 (85.36)	191.25 (76.14)	0.439
RRTri	16.31 (4.95)	12.62 (9.76)	0.019
LF ms^2^	1187.97 (743.46)	556.25 (542.06)	0.001
HF ms^2^	1141.65 (899.22)	572.87 (517.38)	0.006
LF un	49.76 (16.72)	54.54 (14.83)	0.452
HF un	50.23 (16.72)	45.45 (14.84)	0.452
LF/HF	1.24 (0.84)	1.65 (1.71)	0.562
SD1	39.01 (15.43)	23.16 (12.33)	0.000
SD2	85.64 (29.36)	54.41 (25.54)	0.000
SD1/SD2	0.46 (0.15)	0.41 (0.12)	0.469
ApEn	1.46 (0.10)	1.44 (0.11)	0.677

Type 1 DMT1: type 1 diabetes mellitus; SDNN: standard deviation of normal RR intervals in a time interval (ms) RMSSD: square root of the mean of the squares of successive differences between normal RR intervals in a time interval (ms); PNN50: percentage of adjacent RRIs that differ by more than 50ms; TINN: triangular interpolation of RR intervals; RRTri: triangular index; LF: low-frequency component; HF: high frequency component; SD1: standard deviation of the instantaneous RR intervals; SD2: long-term variability of continuous RR intervals; ApEn: approximate entropy.

Table 3 shows sensitivity, specificity, ROC curve, positive predictive value and negative predictive value of HRV. The RMSSD, SDNN, LF ms^2^, HF ms^2^, RRTri, SD1 and SD2 indices showed the best diagnostic accuracy (ROC curve > 0.65).

**Table 3:** Sensitivity, specificity, ROC curve, positive predictive value and negative predictive value for heart rate variability indices

Indices	SEN	SPE	ROC	PPV	NPV
RMSSD	0.66 [0.49 - 0.80]	0.81 [0.66 - 091]	0.79 [0.69 - 0.87]	0.76 [0.58 - 0.89]	0.72 [0.58 - 0.84]
SDNN	0.57 [0.40 - 0.73]	0.88 [0.74 - 0.96]	0.80 [0.70 - 0.88]	0.81 [0.61 - 0.93]	0.70 [0.56 - 0.82]
PNN50	0.71 [0.55-0.85]	0.72 [0.56-0.84]	0.77 [0.66-0.85]	0.70 [0.53-0.83]	0.73 [0.58-0.83]
LF (ms^2^)	0.79 [0.63 - 0.90]	0.69 [0.53 - 0.82]	0.75 [0.64 - 0.84]	0.70 [0.54 - 0.83]	0.75 [0.59 - 0.87]
HF (ms^2^)	0.82 [0.66 - 0.92]	0.55 [0.39 - 0.70]	0.74 [0.63 - 0.83]	0.62 [0.47 - 0.76]	0.77 [0.58 - 0.90]
LF/HF (ms)	0.84 [0.69 - 0.94]	0.32 [0.19 - 0.48]	0.56 [0.45 - 0.67]	0.53 [0.40 - 0.66]	0.70 [0.45 - 0.88]
LF nu	0.84 [0.69 - 0.94]	0.32 [0.19 - 0.48]	0.56 [0.45 - 0.67]	0.53 [0.40 - 0.66]	0.70 [0.45 - 0.88]
HF nu	0.84 [0.69 - 0.94]	0.32 [0.19 - 0.48]	0.56 [0.45 - 0.67]	0.53 [0.40 - 0.66]	0.70 [0.45 - 0.88]
TINN	0.53 [0.37 - 0.69]	0.79 [0.64 - 0.90]	0.63 [0.52 - 0.74]	0.70 [0.50 - 0.85]	0.65 [0.50 - 0.78]
RRTri	0.69 [0.52 - 0.83]	0.76 [0.61 - 0.88]	0.76 [0.65 - 0.85]	0.73 [0.55 - 0.86]	0.73 [0.58 - 0.85]
SD1	0.66 [0.49 - 0.80]	0.79 [0.64 - 0.90]	0.78 [0.68 - 0.87]	0.74 [0.56 - 0.87]	0.72 [0.57 - 0.84]
SD2	0.61 [0.44 - 0.76]	0.88 [0.74 - 0.96]	0.80 [0.70 - 0.88]	0.82 [0.64 - 0.94]	0.71 [0.57 - 0.83]
SD1/SD2	0.46 [0.30 - 0.62]	0.76 [0.61 - 0.88]	0.58 [0.46 - 0.68]	0.64 [0.44 - 0.81]	0.61 [0.46 - 0.74]
ApEn	0.35 [0.21 - 0.52]	0.86 [0.72 - 0.94]	0.56 [0.44 - 0.67]	0.70 [0.45 - 0.88]	0.59 [0.46 - 0.71]

SEN: sensitivity; SPE: specificity; PPV: positive predictive value; NPP: negative predictive value; SDNN: standard deviation of normal RR intervals in a time interval (ms); RMSSD: square root of the mean of the squares of successive differences between normal RR intervals in a time interval (ms); PNN50: percentage of adjacent RRIs that differ by more than 50 ms; TINN: triangular interpolation of RR intervals; RRTri: triangular index; LF: low-frequency component; HF: high-frequency component; nu: normalized unit; SD1: standard deviation of the instantaneous RR intervals; SD2: long-term variability of continuous RR intervals; ApEn: approximate entropy.

Table 4 shows sensitivity, specificity, ROC curve and cutoff point for HRV indices that had a ROC curve > 0.65. Among these indices, the SDNN and SD2 showed the best accuracy.

**Table 4:** Sensitivity, specificity, ROC curve and cutoff points of heart rate variability indices with ROC curve > 0.65

Indices	SEN	SPE	ROC	Cutoff point
RMSSD	0.66 [0.49 - 0.80]	0.81 [0.66 - 091]	0.79 [0.69 - 0.87]	37.00
SDNN	0.57 [0.40 - 0.73]	0.88 [0.74 - 0.96]	0.80 [0.70 - 0.88]	41.90
PNN50	0.71 [0.55-0.85]	0.72 [0.56-0.84]	0.77 [0.66-0.85]	18.50
LF (ms^2^)	0.79 [0.63 - 0.90]	0.69 [0.53 - 0.82]	0.75 [0.64 - 0.84]	711.00
HF (ms^2^)	0.82 [0.66 - 0.92]	0.55 [0.39 - 0.70]	0.74 [0.63 - 0.83]	826.00
RRTri	0.69 [0.52 - 0.83]	0.76 [0.61 - 0.88]	0.76 [0.65 - 0.85]	12.66
SD1	0.66 [0.49 - 0.80]	0.79 [0.64 - 0.90]	0.78 [0.68 - 0.87]	26.20
SD2	0.61 [0.44 - 0.76]	0.88 [0.74 - 0.96]	0.80 [0.70 - 0.88]	55.60

SEN: sensitivity; SPE: specificity; RMSSD: square root of the mean of the squares of successive differences between normal RR intervals in a time interval (ms); SDNN: standard deviation of normal RR intervals in a time interval (ms); LF: low-frequency component; HF: high-frequency component; RRTri: triangular index; SD1: standard deviation of the instantaneous RR intervals; SD2: long-term variability of continuous RR intervals.

## Discussion

Our findings indicate that individuals with type 1 DM have altered HRV, characterized by a reduction in sympathetic and parasympathetic activities, and in overall variability as compared with healthy controls. In addition, the RMSSD, SDNN, PNN50, LF ms^2^, HF ms^2^, RRTri, SD1 and SD2 indices had the best diagnostic accuracy in discriminating type 1 diabetic patients from healthy individuals.

Also, type 1 DM subjects had higher body mass, BMI, random glucose levels, and percentage of body fat compared with healthy volunteers, whereas the variables age, height, WHR, SAP, DAP and physical activity were not different between the groups. Similar results were reported by Javorka et al.^32^ for age, BMI, SAP and DAP, and by Jaiswal et al.^12^ for HR and physical activity.

The HRV results indicated a decrease in sympathetic (LF ms^2^) and parasympathetic activities (RMSSD, PNN50, HF ms^2^), and in overall variability (SDNN, RRtri and SD2) in type 1 DM subjects as compared with healthy controls. These findings are corroborated by Javorka et al.^9^, who reported a decrease in SDNN, RMSSD, PNN50, LF ms^2^ and HF ms^2^ in 17 type 1 DM subjects (22.4 ± 1.0 years). Jaiswal et al.,^12^ in a study on more than 350 young individuals (18.8 ± 3.3 years with type 1 DM), observed significantly lower SDNN, RMSSD, HF nu, LF nu and LF/HF ratio in this population than in healthy controls. However, in our study, no differences in SD1/SD2, LF/HF and LF and HF in normalized units were observed between the groups.

Changes in HRV are indicative of abnormal, insufficient adaptation of ANS,^7^ which increases the risk of sudden death for heart arrhythmias, and is associated with increased mortality rate for other causes.^33^ This indicates that the cardiovascular autonomic dysfunction may be a complicating factor in patients already at risk, as in DM patients.^34^

Other studies demonstrated that some HRV indices have good diagnostic accuracy in some populations.^15-17^ In our study, the RMSSD, SDNN, PNN50, LF ms^2^, HF ms^2^, RRtri, SD1 and SD2 indices showed greater sensitivity and specificity to detect autonomic dysfunction in type 1 DM patients and in healthy individuals. Indices with higher discriminatory power were those with significantly lower values in the type 1 DM group than in the control group.

These indices are associated with the analysis of parasympathetic activity (RMSSD, PNN50, HF ms^2^ and SD1), sympathetic activity (LF ms^2^) and overall ANS behavior (SDNN, RRtri and SD2),^7^ suggesting that discrimination of patients with type 1 DM may be related to the reduction in autonomic, global and sympathetic modulation of the heart.

Few studies have evaluated the diagnostic power of HRV in type 1 DM. Ziegler et al.^35^ have shown that the HF index showed greater sensitivity to detect early autonomic dysfunction in type 1 and type 2 DM patients classified in the three stages of cardiac autonomic neuropathy. Khandoker et al.^15^ found that the SampEn and the SD1/SD2 ratio, obtained from the Poincaré plot, were better discriminators of type 1 or type 2 DM patients with cardiac autonomic neuropathy, with a 100% sensitivity and 75% specificity.

Takase et al.^36^ demonstrated that SDANN lower than 30ms had greater sensitivity (72%) and specificity (92%) than SDANN higher than 20 ms (31% sensitivity and 100% specificity) to detect autonomic dysfunction and cardiac events in type 2 DM patients with cardiac autonomic neuropathy.

Nonetheless, in these studies,^35,36^ only patients with established cardiac autonomic neuropathy were included, except for the study by Khandoker et al.,^15^ that evaluated diabetic individuals, regardless of the diagnosis of neuropathy. In our study, diagnostic accuracy of HRV was analyzed in both groups (DM and control) at the same time, aiming to evaluate the power to discriminate type 1 DM subjects from controls by the presence of changes in cardiac autonomic modulation, providing results that are closer to the clinical practice.

Therefore, a strength of the study is that the capacity of HRV to diagnose possible autonomic changes were assessed in type 1 DM individuals, resulting in a cutoff value that provides evidence to healthcare professionals for changes that may be associated with early cardiac autonomic neuropathy. It is worth mentioning that none of the volunteers had cardiac autonomic neuropathy as a complication of type 1 DM. For this reason, different from previous studies,^15,35,36^ we cannot affirm that the cutoff values identified in this study are associated with this condition, but rather with an ANS depression possibly related to the type 1 DM,^8-14^ that should be investigated and treated, to prevent the progression to cardiac autonomic neuropathy. Also, it is worth mentioning that a decrease in HRV is the first sign of autonomic neuropathy and suggested as one of the diagnostic tests in a statement by the American Diabetes Association's position statement.^6^

The validity of a test refers to its capacity in diagnosing or predicting an event, and the values of sensitivity and specificity give the probability of a test to correctly discriminate an individual with a disease from a healthy individual,^37^ hence reducing the risk of an erroneous diagnosis. In our study, 8 of the 14 indices tested showed greater sensitivity and specificity in discriminating type 1 DM individuals from those without the disease, and their use as diagnostic tools may be encouraged.

HRV analysis is a fast, safe, non-invasive and financially accessible method, which enables the clinical follow-up of ANS condition. This is essential to reduce and intervene in case of complications to reduce cardiovascular events,^33^ sudden death,^38^ and loss of quality of life^39^ in this population.

One limitation of this study is its cross-sectional design that prevented us to evaluate the autonomic behavior for a longer period and conclude whether the changes in ANS condition, detected in the study, were in their initial stage or not. In addition, the fact the group of diabetic patients had different time of diagnosis, and that this group had greater mean BMI and body fat percentage than the control group may have influenced the results. Longitudinal studies are needed to confirm whether these indices with the best discriminatory power maintain their prognostic capacity in long term.

## Conclusion

type 1 DM patients have autonomic changes characterized by reduction in sympathetic and parasympathetic activities and overall variability. The SDNN, RMSSD, PNN50, RRtri, LF ms^2^, HF ms^2^, SD1 and SD2 indices had the best diagnostic accuracy in discriminating individuals with type 1 DM.
